# Impact of exposure to diesel exhaust during pregnancy on mammary gland development and milk composition in the rabbit

**DOI:** 10.1371/journal.pone.0212132

**Published:** 2019-02-14

**Authors:** Cathy Hue-Beauvais, Etienne Aujean, Guy Miranda, Delphine Ralliard-Rousseau, Sarah Valentino, Nicolas Brun, Stessy Ladebese, Christine Péchoux, Pascale Chavatte-Palmer, Madia Charlier

**Affiliations:** 1 UMR GABI, INRA, AgroParisTech, Université Paris-Saclay, Jouy-en-Josas, France; 2 UMR BDR, INRA, ENVA, Université Paris Saclay, Jouy-en-Josas, France; Telethon Institute for Child Health Research, AUSTRALIA

## Abstract

Exposure to fine-particulate air pollution is a major global health concern because it is associated with reduced birth weight and an increased risk of cardiovascular disease. Here we have investigated the potential for exposure to diesel exhaust during pregnancy to influence mammary gland development and milk composition. Female rabbits were therefore exposed by nose-only inhalation to either diluted diesel exhaust fumes (1 mg/m^3^) or clean air for 2h/day, 5 days/week, from the 3^rd^ to the 27^th^ days of pregnancy. On Day 28 of pregnancy, mammary glands were collected from twelve females (six controls and six diesel-exposed) and assessed for morphological and functional alterations. Milk samples were collected from eighteen dams (nine controls and nine diesel-exposed) during early (days 2 to 4) and established (days 13 to 16) lactation to verify the composition of fatty acids and major proteins and leptin levels. The mammary alveolar lumina contained numerous fat globules, and stearoyl CoA reductase expression was higher in mammary epithelia from diesel exhaust-exposed rabbits, which together suggested increased mammary lipid biosynthesis. Gas chromatography analysis of the composition of milk fatty acids revealed a sharp rise in the total fatty acid content, mainly due to monounsaturated fatty acids. Liquid chromatography-mass spectrometry analysis of milk samples enabled identification and quantification of the main rabbit milk proteins and their main phosphorylated isoforms, and revealed important changes to individual casein and whey protein contents and to their most phosphorylated isoforms during early lactation. Taken together, these findings suggest that repeated daily exposure to diesel exhaust fumes during pregnancy at urban pollution levels can influence lipid metabolism in the mammary gland and the lipid and protein composition of milk. As milk may contribute to metabolic programming, such alterations affecting milk composition should be taken into account from a public health perspective.

## Introduction

Diesel engine exhaust (DE) is an environmental risk factor for humans since it has been linked to various deleterious effects such as increased morbidity and mortality due to air pollution [1, 2] an increased risk of lung cancer [3] and respiratory diseases [4], reproductive disorders [5, 6] and genotoxicity affecting various tissues [7, 8]. Diesel engine exhaust is a complex mixture of gaseous and particulate components. The particles released by DE are usually fine, with an aerodynamic diameter ≤2.5 μm, as well as a very high proportion of ultrafine particles with a diameter of less than 0.1 μm, which are probably more harmful than the larger fraction [9]. In the environment, they combine very rapidly into larger groups and aggregate. Because the particles are very small, their overall surface is very large, thus enabling the adsorption of a variety of compounds.

The mammary network develops over a long period of time, being initiated during foetal life and continuing until the first lactation in adulthood. During pregnancy, the mammary gland develops to become a branched epithelial network of ducts that support large alveolar development and allow subsequent milk production during lactation [10]. Mammary epithelial growth and differentiation are tightly modulated throughout development by several hormonal and metabolic signals [11], suggesting the existence of critical periods during which the mammary gland is vulnerable to developmental programming by environmental stressors, as explained in the developmental origins of health and diseases (DOHaD) concept [12, 13].

An increasing number of studies in both human and animal models have shown that prenatal exposure to ambient fine particles is associated with a variety of abnormalities during foetal development, such as preterm birth, low birth weight and placental dysfunctions [14–17]. Moreover, there is clear evidence that gestational exposure to pollutants can also have deleterious long term effects that mainly involve energy metabolism disorders [18, 19] and cardiac and respiratory dysfunctions [4, 20, 21]. We have previously reported that females exposed daily during pregnancy to DE particles at levels close to urban pollution evidenced foeto-placental disorders [14]. Rabbits were used for these studies as they can be considered as a relevant model for human physiology due to their similarities in lipid metabolism [22] and in placentation closer to humans than that of rodents [23] as well as because the rabbit-primate phylogenetic distance is the same as the rodent-primate distance [24]. Moreover, because rodent sequences have evolved more rapidly, rabbit gene sequences are more similar to those of humans than to those of rodents, which might be of considerable importance to further analyses of altered gene expression profiles [24].

Since early postnatal life is considered to be a period that is critical to the long-term health and disease susceptibility of offspring, the impact of pollutants on mammary gland development and subsequent milk composition is of crucial importance. Rabbit milk is characterized by a high fat and protein content, an absence of β-lactoglobulin, the presence of whey acidic protein (WAP), the existence of a second α_s2_ casein (α_s2_-like-casein) and a high level of lactoferrin [25, 26]. As milk was shown to exert effects far beyond its immediate nutritional value, the objective of the present study was therefore to determine the effects of maternal exposure to DE during pregnancy on mammary gland development in terms of tissue morphology and function. In addition, we investigated the impact of DE exposure on milk composition, including the fatty acid content and relative proportions of the main milk proteins. Furthermore, because leptin plays a key role in regulating developmental programming [27], we examined the leptin levels in mammary tissue and different milk samples in order to link maternal DE exposure to a potential for subsequent risks of growth and metabolic disorders in their offspring.

## Materials and methods

### Ethics statement

All animal studies were performed in compliance with European Community regulations on animal experimentation (European Communities Council Directive 86/609/EEC; European Union, 1986) and with the authorisation of the French Ministry of Agriculture. All protocols were approved (visa 12–102) by the Comité d’Ethique en Expérimentation Animale (Ethics Committee on Animal Experimentation), registered as N°C2EA-45 by the French National register CNREEA (Comité National de Reflexion Ethique sur l’Expérimentation Animale by the Comité National de Réflexion Ethique sur l’Expérimentation Animale, French National Ethics Committee on Animal Experimentation).

### Animal experiments and sample collection

The rabbits used during this study were the same as those described in detail elsewhere [14]. Briefly, one year old female rabbits (New Zealand White, 1077-INRA) were housed individually in an indoor facility under controlled light conditions (8 h light/16 h darkness except from the week before mating when they were under 16 h light/8 h darkness) and temperature (18°C). In their first pregnancy rabbits were exposed to diluted (1 mg/m^3^) and filtered diesel engine (DE) exhaust fumes (nanoparticle diameter ~ 69 nm) or clean air (controls) for 2h/day, 5 days/week by means of nose-only exposure from the 3^rd^ to the 27^th^ days post-conception (total exposure of 20 days out of a 31-day pregnancy) as previously described [14]. DE exposure was performed with the Mobile Ambient Particle Concentrator Exposure Laboratory [28] connected to a 25KVA Loxam engine, with a 500 nm particle filter. DE is a complex mixture of hundreds of constituents in either a gas or particle form. Gaseous components of DE include carbon dioxide, oxygen, nitrogen, water vapour, carbon monoxide, nitrogen compounds, sulphur compounds, and numerous low-molecular-weight hydrocarbons (some of them individually known to be toxic, such as aldehydes, benzene, 1,3-butadiene, polycyclic aromatic hydrocarbons (PAHs) and nitro-PAHS). The particles present in DE are known to be composed of center core of elemental carbon with absorbed organic compounds and small amounts of sulphate, nitrate, metals, and other trace elements [29].

On Day 28 of pregnancy, before parturition, 12 dams (six exposed to DE and six controls (C)) were sacrificed by exsanguination after electronarcosis and the left lower mammary gland from each animal was excised and dissected. As fetal organs were collected for further examination, fetuses were euthanised by decapitation after removal of the uterus in accordance with the Ethics Committee on Animal Experimentation as well as the French Ministry of Agriculture (visa 12–102). Dams’ mammary epithelial tissue was either processed for histology or snap-frozen and stored for later RNA isolation.

Milk samples were collected by hand stripping from 18 other females dedicated to post-natal follow-up of the offspring (nine exposed to DE and nine C) on days 2–4 and days 13–16 of lactation, as previously described [30]. The samples thus collected were stored at -20°C until analysis. Two groups were thus formed and named as a function of the prior exposure of the dams during pregnancy (C milk and DE milk, respectively).

### Histological and ultrastructural analyses of mammary tissue

For histological analyses, mammary samples were fixed in 4% paraformaldehyde, cryoprotected in 40% sucrose and embedded in TissuTek (Sakura, Torrance, CA). Five micrometre sections, at least 100 μm apart, were mounted on slides, stained with hematoxylin and eosin (H&E; Sigma-Aldrich, Saint Quentin Fallavier, France) and digitised using a Hamamatsu NanoZoomer (Hamamatsu Photonics, Tokyo, Japan). Five sections per rabbit were processed and the images were analysed using both the Hamamatsu NanoZoomer Digital Pathology Virtual Slide Viewer and ImageJ software [31]. The areas occupied by mammary epithelial tissue, adipose tissue, mammary ducts or connective tissue were measured.

For transmission electron microscopy, mammary tissue was fixed with 2% glutaraldehyde in 0.1M Na cacodylate buffer pH 7.2 for 4 hours at room temperature. The samples were then contrasted with 0.5% Oolong Tea Extract (OTE) in 0.1M Na cacodylate buffer, post-fixed with 1% osmium tetroxide containing 1.5% potassium cyanoferrate, gradually dehydrated in ethanol (30% to 100%) and gradually substituted in a mix of propylene oxyde-epon and embedded in Epon. (Delta Microscopie, Labège, France). Half-micron sections were collected onto glass slides, counterstained with methylene blue-Azur II and imaged under an optical microscope.

The surface occupied by luminal fat globules was measured in relation to the total area of the lumen, using a threshold method with the Fiji module of ImageJ software. A total of 88 lumina for C mammary glands and 108 lumina for DE-exposed mammary glands were processed.

### Immunolocalisation of leptin in the mammary gland

Leptin was localised by immunohistochemical analyses using a 1:100 dilution of goat anti-rat leptin antibody (AbD Serotec, France). Briefly, 5 μm sections were treated in 50 mM ammonium chloride for 30 min followed by permeabilisation in 2% BSA, 0.05% saponin and 0.05% sodium azide in PBS 1X for 1 h. The tissue sections were then incubated overnight at 4°C with primary antibody. Antibody binding was visualised after incubation for 45 min with fluorescence-labelled anti-goat TRITC-conjugated antibody (dilution: 1:300, Jackson Immunoresearch). The slides were then mounted in Vectashield mounting medium (Vector Laboratories, Burlingame, CA) and observed with a Zeiss microscope coupled with ApoTome technology.

### Reverse transcription and quantitative PCR

For gene assays in the mammary gland, reverse transcription was performed on 500 ng of total RNA using the SuperScript1 VILO cDNA Synthesis kit according to the manufacturer’s instructions (Invitrogen, France) and under the following conditions: 42˚C for 60 min and 85˚C for 5 min. Quantitative PCR runs were achieved using the ABsolute Blue QPCR Mix, SYBR Green1 (Thermo Scientific, France) according to the manufacturer’s instructions, on a Mastercycler Eppendorf Realplex system (Eppendorf, France) under the following conditions: 95˚C for 15 min, 45 cycles of 95˚C for 15 sec and 60˚C for 1 min, and a melting curve.

The threshold cycles obtained for Leptin (F 5’-ACACACGCAGTCGGTCTC-3’ and R 5’- GAGGTTCTCCAGGTCGTTGG-3’), SREBP1 (Sterol Regulatory Element-Binding Protein 1, F 5’- CCAGCTGACAGCTCCATTGA-3’ and R 5’- TGCGCGCCACAAGGA -3’), FASN (Fatty Acid Synthase N, F 5’-ACCTCGTGAAGGCTGTGACTCA-3’ and R 5’-TGAGTCGAGGCCAAGGTCTGAA-3’), SCD (Stearoyl-CoA Desaturase, F 5’-TTATTCCGTTATGCCCTTGG-3’ and R 5’-TTGTCATAAGGGCGGTATCC-3’) and LPL (LipoProtein Lipase, F 5’-CTCAGGACTCCCGAAGACAC-3’ and R 5’-GTTTTGCTGCTGTGGTTGAA-3’) were normalised with the values of TBP (TATA Binding Protein F 5’-TGACCCCCATGACCCCTATT-3’ and R 5’-CAGCAAACCGCTTGGGATTA-3’) and the results were expressed as fold changes of the threshold cycle (Ct) values relative to the control using the 2-ΔΔCt method [32].

### Analysis of the milk fatty acid composition

Analyses were performed on 18 individual milk samples from DE and C dams (9 C and 9 DE), collected on days 2–4 (L2-4) and days 13–16 (L13-16) of lactation. To determine the fatty acid composition, 200-μl full-fat milk were added to an internal standard (margaric acid, C17:0) before lipid extraction with chloroform/methanol (2:1, adapted from the method described by Folch et al. [33]). The fatty acids were transmethylated with 7% boron trifluoride methanol (Sigma-Aldrich, Saint Quentin Fallavier, France) in accordance with the method previously described by Morrison and Smith [34]. Finally, the methyl esters of milk fatty acids were analysed using gas chromatography (Auto Sampling 8410 Gas Chromatograph 3900; Varian, Les Ulis, France) coupled to a flame ionisation detector on an Econo-Cap EC-WAX capillary column (30 m, 0.32-mm internal diameter, 0.25-μm film, reference 19654; ALLTECH Associates Inc., Templemars, France), as described by Rousseau et al.[35]. Identification of FA was made in reference to known FA profiles obtained after injection of standard FAME (FA methyl esters) mix (Supelco 37 components FAME mix, ref 47885-U, Sigma). The fatty acids profile was established for each sample and expressed as percentage of total FA. Heptadecanoic acid (C17:0), introduced prior to milk lipid extraction (50μg) was used as an internal standard to measure the total FA concentration after chromatogram analysis.

### Analysis of the main proteins in milk

Analyses were performed on the same 18 individual milk samples from DE and C dams. The milk samples were skimmed by centrifugation at 2000 × *g* for 20 min at 4°C, then analysed as previously described [36] using reverse-phase high-performance liquid chromatography (HPLC) coupled with electrospray ionisation (ESI) mass spectrometry. Peak profiles from UV absorbance at 214 nm were analysed using Chromeleon software (Chromeleon 7.0.0; Dionex, Thermo Fisher Scientific). Protein isoforms of the principal milk proteins were identified by comparing their molecular masses with an in-house mass database on rabbit milk proteins and the UniProt database (UniProt Consortium, 2015; http://www.uniprot.org [Accessed July 2015]). Moreover, the main milk proteins were quantified by integrating the chromatogram peak areas at 214 nm. Values were expressed as a percentage of the total peak area.

### Measurement of leptin concentrations in milk

Quantification of the levels of rabbit leptin in each milk sample was performed using an ELISA kit (Cloud Clone Corp., Houston, TX) according to the manufacturer’s instructions. Triplicates of each milk sample were used and the results reported here represent the mean of these three measurements.

### Statistical analysis

The data were analysed using a nonparametric Mann-Whitney U-test and significant differences were defined as P<0.05. When necessary, statistical analysis was supplemented by an ANOVA approach.

## Results

### Maternal characterisation

Exposed females exhibited no alteration either concerning their plasma fatty acid profile or biochemical data. Moreover, within pups, sex ratio or litter size were similar among groups [14].

### DE Exposure throughout pregnancy does not alter mammary gland histology but increases the alveolar fat globule content

**Fig 1:**
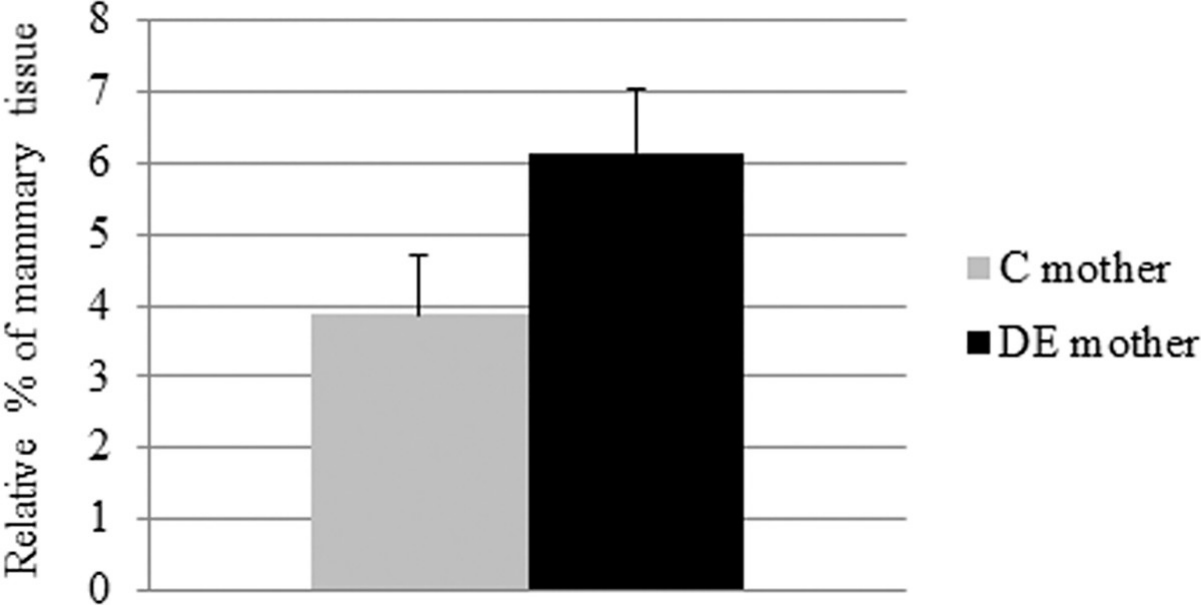
Quantification by stereology of the surface occupied by mammary ducts’ and alveoli’s lumina. Analyses were performed in C and DE-exposed rabbits at Day 28 of pregnancy using the Hamamatsu NanoZoomer Digital Pathology Virtual Slide Viewer and ImageJ software. Data are mean ± SEM.

Histological examinations were performed to determine whether DE exposure during pregnancy induced morphological changes to the mammary gland. Morphological differences were quantitatively analysed based on the relative ratio of the surfaces of mammary ducts and alveoli’s lumina. These analyses did not reveal any significant differences between DE and control animals on Day 28 of pregnancy (Fig 1).

**Fig 2:**
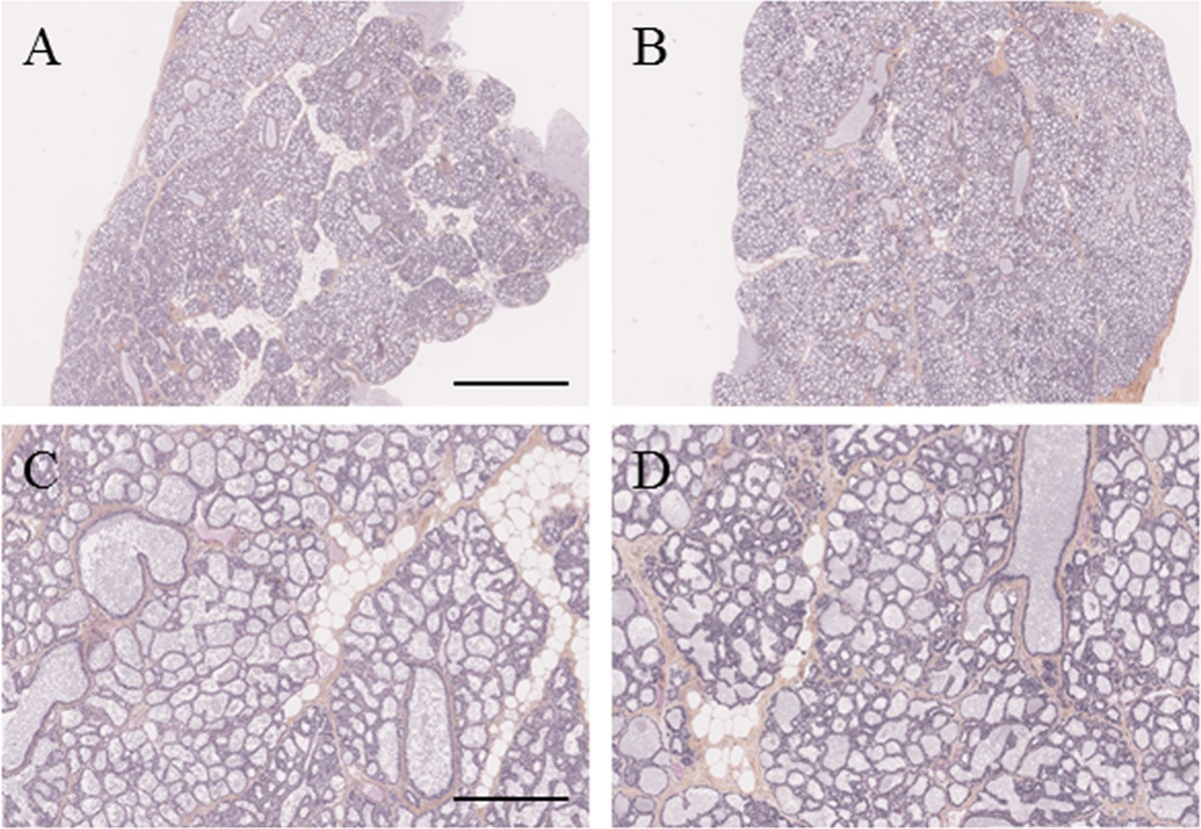
Histological sections of mammary gland from C and DE rabbits at Day 28 of pregnancy. Hematoxylin and eosin staining of 5μm cryosections of mammary glands from C (A and C) and DE-exposed (B and D) rabbits were observed at different magnifications. Representative nanozoomer scans were observed at low magnification (A and B: the scale bar represents 2 mm) and higher magnification (C and D: the scale bars represent 250 μm).

To further investigate the alveolar content, thin mammary tissue sections were performed in control (Fig 2A) and DE (Fig 2B) dams, and the relative surface area occupied by fat globules was measured in the alveolar lumina. A clear modification was observed in the alveolar lumina of DE rabbits. Indeed, all DE mammary glands were characterised by a significant increase in the total surface area occupied by fat globules (23.80 ± 9.37% *vs*. 38.72 ± 2.40%, p = 0.008) as the representative shown in Fig 2C and 2D.

### DE Exposure during pregnancy does not modify leptin expression and protein localisation in the mammary gland

**Fig 3:**
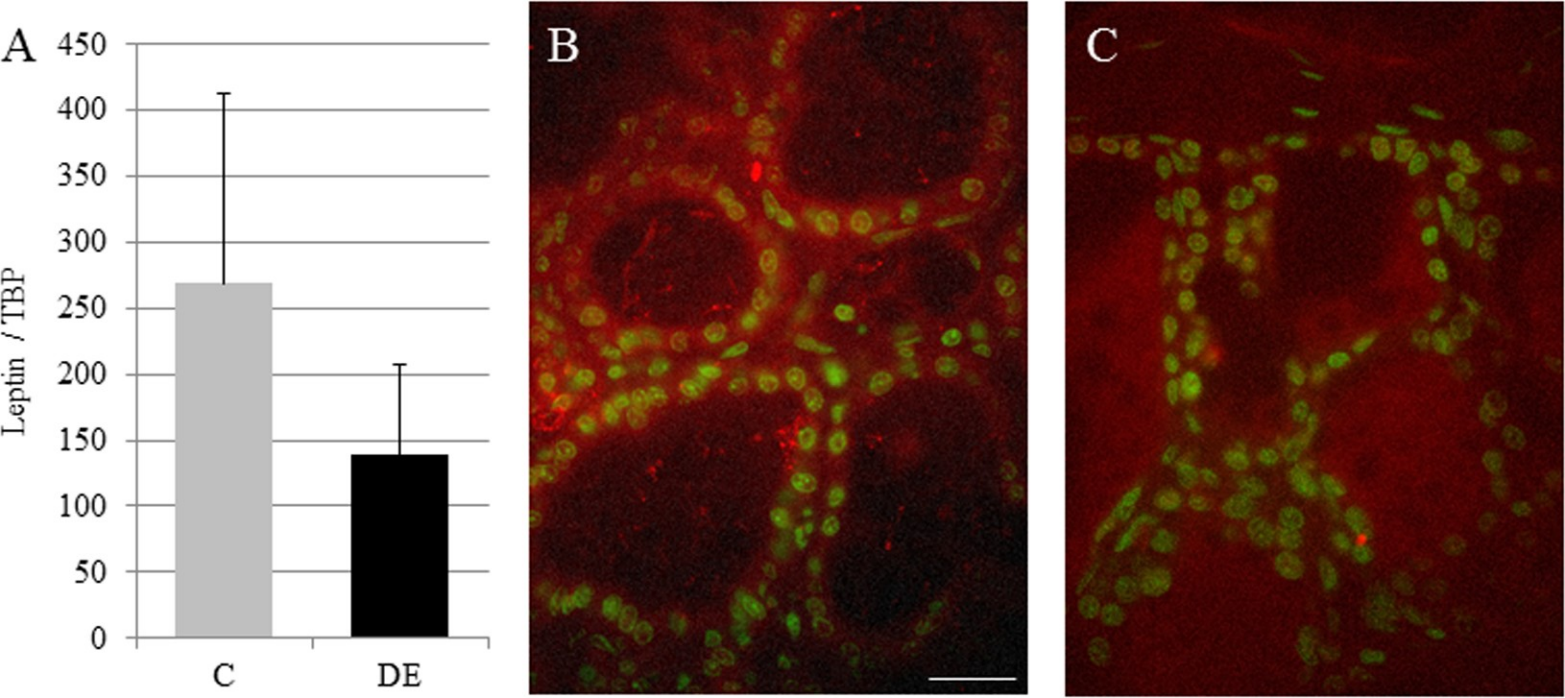
Leptin expression and localisation in mammary glands from C and DE rabbits at day 28 of pregnancy. (A) Expression of leptin in mammary glands at day 28 of pregnancy from control (in light grey) and exposed (DE) (in black) dams. Real-time RT-PCR was quantified using TBP housekeeping gene. Data are mean ± SEM. (B and C) Representative images of leptin immunostaining (red) of mammary gland from control (B) or exposed (C) dams at day 28 of pregnancy. Nuclei are stained in green. Scale bar represents 50μm.

To examine the expression of leptin mRNA and protein localisation in the mammary glands of C and DE-exposed dams on Day 28 of pregnancy, RT-qPCR analyses and immunostaining experiments were performed (Fig 3). No significant differences were observed between the two groups of animals with respect to mRNA expression, although there were some signs of a decrease in DE mammary glands. Immunolocalisation studies showed that the epithelial localization of leptin in mammary tissue was not altered following DE exposure.

### Impact of DE Exposure on the Expression of the *LPL*, *FASN*, *SREBP* and *SCD* Genes Involved in Lipid Metabolism in the mammary gland

**Fig 4:**
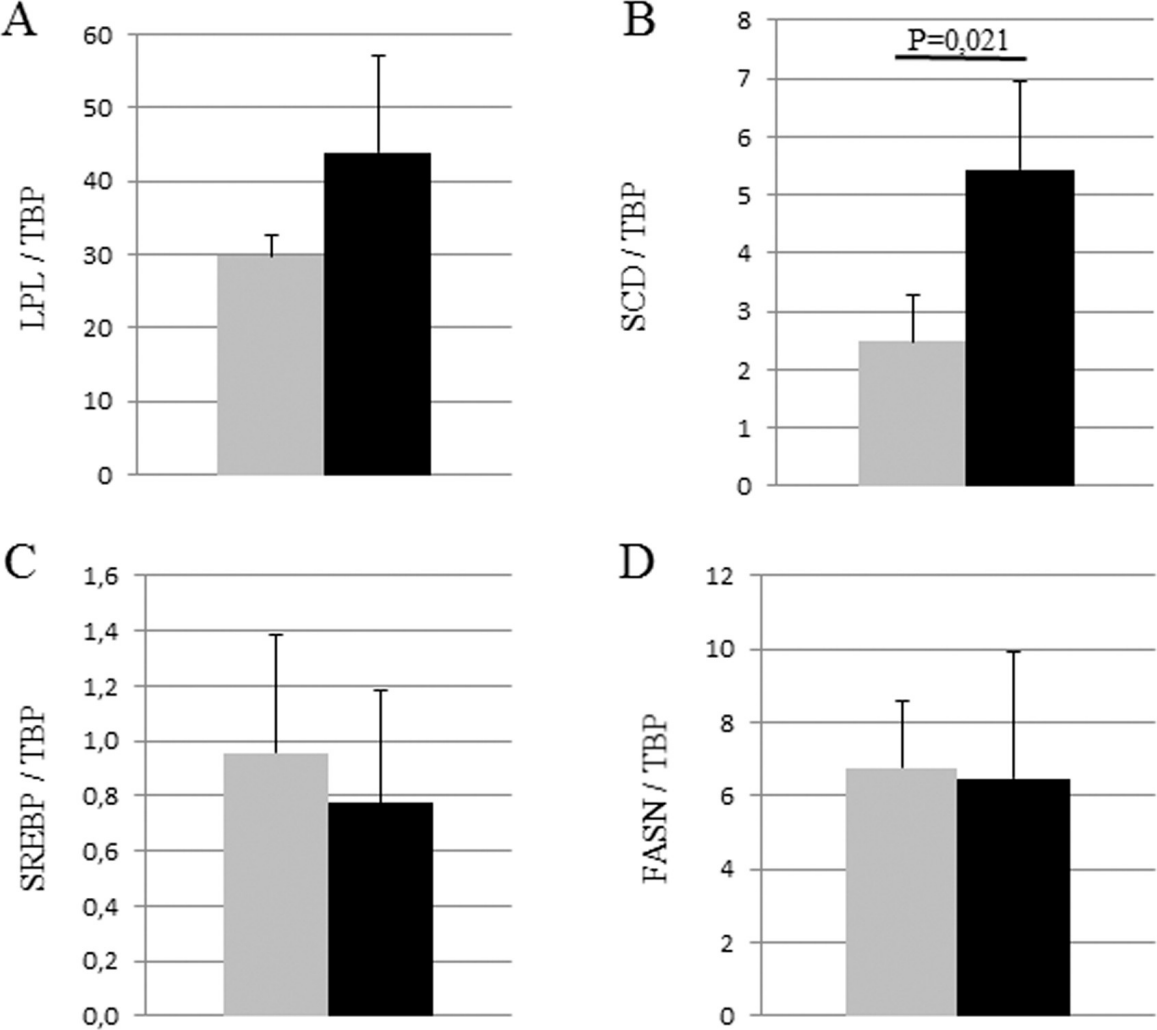
Expression of lipid metabolism enzymes in the mammary glands of C and DE rabbits determined using real-time RT-PCR. **(**A) Lipoprotein lipase (LPL), (B) Stearoyl CoA desaturase (SCD), (C) Sterol regulatory element-binding protein (SREBP) and (D) Fatty Acid Synthase N. Expression was measured in mammary tissue from control (in light grey) and DE exposed (black) dams, at 28 days of pregnancy. Data are mean ± SEM.

To determine whether DE exposure might affect the expression of key genes involved in lipid metabolism, RT-qPCR experiments were performed on total mammary RNA (Fig 4). Among the four genes tested, only SCD was significantly increased in DE animals compared to controls (SCD/TBP ratio: 2.48 ± 0.80 *vs*. 5.43 ± 1.54, p = 0.021), suggesting increased fatty acid synthesis in the mammary gland of DE rabbits on Day 28 of pregnancy.

### DE exposure alters the fatty acid profile of milk

To evaluate DE exposure-induced alterations to milk composition, fatty acid profiles were determined in milk from C and DE-exposed dams at two stages of lactation: early (2–4 days, L2-L4) and mid (13–16 days, L13-L16) lactation (Tables 1 and 2).

**Table 1:** Fatty acid composition and content in milk from control (C) and DE-exposed (DE) dams on days 2–4 of lactation.

Fatty acids	C milk^1^	DE milk^1^	C milk^2^	DE milk^2^
C8:0	17.77 (0.85) ^a^	15.83 (0.73) ^a^	13.32 (0.66) ^a^	15.57 (0.88) ^a^
C10:0	19.99 (1.00)	18.76 (0.80)	14.96 (0.70) ^a^	18.81 (1.12) ^a^
C12:0	3.74 (0.31)	3.68 (0.29)	2.80 (0.23) ^a^	3.79 (0.33) ^a^
C14:0	1.94 (0.06)	1.88 (0.10)	1.46 (0.10) ^a^	1.92 (0.15) ^a^
C16:0	15.49 (0.63) ^a^	16.79 (0.51) ^a^	11.71 (0.90) ^a^	16.76 (1.26) ^a^
C18:0	3.21 (0.25)	2.89 (0.12)	2.43 (0.25)	2.88 (0.23)
**SFA**	**62.67 (1.17)**	**60.30 (1.07)**	**47.08 (1.92)** ^a^	**60.20 (3.44)** ^a^
**sc SFA**	**41.51 (1.77)**	**38.27 (1.49)**	**31.07 (1.28)** ^a^	**38.17 (2.17)** ^a^
**mlc SFA**	**21.16 (0.87)**	**22.03 (0.58)**	**16.00 (1.25)** ^a^	**22.03 (1.63)** ^a^
C16:1 n-9	0.26 (0.01) ^a^	0.32 (0.02) ^a^	0.20 (0.01) ^a^	0.32 (0.02) ^a^
C18:1 n-9	16.42 (0.50)	17.39 (0.53)	12.37 (0.75) ^a^	17.24 (1.16) ^a^
**MUFA**	**19.56 (0.68)**	**20.96 (0.79)**	**14.74 (0.97)** ^a^	**20.90 (1.49)** ^a^
C18:2 n-6	14.72 (0.48)	15.16 (0.48)	11.10 (0.73) ^a^	15.00 (1.12) ^a^
C18:3 n-3	2.15 (0.05)	2.10 (0.05)	1.62 (0.10) ^a^	2.09 (0.15) ^a^
**PUFA**	**17.77 (0.52)**	**18.74 (0.56)**	**13.40 (0.84)** ^a^	**18.49 (1.20)** ^a^
n-6 PUFA	15.57 (0.48)	16.18 (0.52)	11.74 (0.75) ^a^	15.99 (1.22) ^a^
n-3 PUFA	2.20 (0.05)	2.18 (0.05)	1.66 (0.10) ^a^	2.19 (0.16)^a^
n-6/n-3 ratio	7.07 (0.13)	7.43 (0.21)		
**Total FA**	100	100	75.22 (3.32) ^a^	99.59 (5.70) ^a^

^1^Percentage of total fatty acids.

^2^Grams per litre.

^a^ P values <0.05 calculated using the Mann-Whitney test to compare the C and DE groups.

**Table 2:** Fatty acid composition and content in milk from control (C) and DE-exposed (DE) dams on days 13–16 of lactation.

Fatty acids	C milk^1^	DE milk^1^	C milk^2^	DE milk^2^
C8:0	25.56 (0.93)	26.06 (0.84)	18.59 (1.86)	21.44 (1.10)
C10:0	20.40 (0.64) ^a^	22.66 (0.31) ^a^	15.13 (1.81)	18.73 (1.03)
C12:0	2.47 (0.15) ^a^	2.97 (0.15) ^a^	1.87 (0.28)	2.47 (0.20)
C14:0	1.47 (0.08)	1.52 (0.13)	1.11 (0.15)	1.27 (0.14)
C16:0	11.71 (0.90)	12.44 (0.65)	9.74 (1.26)	10.41 (1.00)
C18:0	3.05 (0.13) ^a^	2.62 (0.10) ^a^	2.26 (0.29)	2.17 (0.16)
**SFA**	**66.44 (0.86)** ^a^	**68.64 (0.47)** ^a^	**48.99 (5.38)**	**56.82 (3.27)**
**sc SFA**	**48.43 (1.44)**	**51.68 (0.80)**	**35.59 (3.91)**	**42.64 (2.17)**
**mlc SFA**	**18.02 (0.68)**	**16.96 (0.74)**	**13.40 (1.71)**	**14.17 (1.27)**
C16:1 n-9	0.20 (0.01)	0.32 (0.02)	0.19 (0.02)	0.23 (0.03)
C18:1 n-9	15.20 (0.42)	14.83 (0.22)	11.33 (1.42)	12.28 (0.77)
**MUFA**	**17.83 (0.50)**	**17.35 (0.31)**	**13.24 (1.62)**	**14.38 (0.93)**
C18:2 n-6	13.05 (0.38) ^a^	11.54 (0.22) ^a^	9.71 (1.22)	9.52 (0.50)
C18:3 n-3	1.80 (0.02)	1.73 (0.03)	1.34 (0.16)	1.43 (0.07)
**PUFA**	**15.73 (0.40)** ^a^	**14.01 (0.25)** ^a^	**11.70 (1.45)**	**11.56 (0.63)**
n-6 PUFA	13.90 (0.39) ^a^	12.23 (0.23) ^a^	10.33 (1.28)	10.09 (0.56)
n-3 PUFA	1.83 (0.03)	1.78 (0.03)	1.36 (0.17)	1.47 (0.08)
n-6/n-3 ratio	7.60 (0.18) ^a^	6.86 (0.12) ^a^		
**Total FA**	100	100	73.94 (8.32)	82.75 (4.75)

^1^Percentage of total fatty acids.

^2^Grams per litre.

^a^ P values <0.05 calculated using the Mann-Whitney test to compare the C and DE groups

During early lactation (L2-L4), the total fatty acid content was higher in DE milk than in C milk (99.6 ± 5.7 g/L and 75.2 ± 3.3 g/L, *P*<0.05, respectively). Quantitatively, DE milk displayed a higher total SFA content than C milk. This rise was due to all SFA except stearic acid (C18:0) which concentration did not vary significantly. Similarly, a sharp increase in the MUFA content was observed in DE milk, alongside a corresponding rise in palmitoleic and oleic acids (*P*<0.05). Polyunsaturated fatty acid levels were also higher in DE milk, with increases in both α-linolenic and linoleic acids (*P*<0.05) although there was no change to the PUFA *n-6*: *n-3* ratio (Table 1). Qualitatively, SFA were the main fatty acids present in both DE and C milks (Table 1). Both short chain and long chain SFA levels increased in DE milk (*P*<0.05). Among MUFA, only palmitoleic acid was present at a higher level in DE milk (0.32 ± 0.02%) than in C milk (0.26 ± 0.01%).

At mid-lactation (L13-16), no quantitative differences in the fatty acid content were observed between C and DE milks. In contrast, a slight qualitative rise in SFA levels (3%) was seen in DE milk, resulting from an increase in C10:0 and a fall of C18:0 SFA (Table 2). No qualitative modifications were observed in the proportions of MUFA, but the PUFA content was reduced in DE milk and led to a decrease in the *n-6*: *n-3* ratio.

Thus, at both stages of lactation, exposure to diesel exhaust throughout pregnancy altered the fatty acid composition, although mainly during early lactation.

### The composition of the main proteins is altered in DE milk

To investigate the impact of DE exposure during pregnancy on the composition of the main milk proteins, LC-MS technology was used to analyse milk samples at two stages of lactation. A qualitative comparison of the chromatogram profiles of milk from DE-exposed dams on Days 2–4 and 13–14 of lactation (S1 Fig) did not reveal any differences regarding the presence of the main milk proteins, nor their variants or phosphorylated isoforms [36].

A quantitative estimate of variations in the peak areas was performed as a function of the relative proportions of whole milk proteins; i.e. the percentage of the total peak areas on the UV chromatogram at 214 nm (Table 3). No variations in the total casein ratio were observed at either the L2-L4 or L13-L16 days of lactation in DE *versus* C milk. However, among the casein proteins, a significant increase in α_s2_-like proteins (*P* <0.05) and a significant decrease in α_S1_ proteins (*P*<0.05) were observed. As for total whey proteins, the proportions of both lactoferrin and serum albumin had risen in DE milk at days L2-L4 (*P*<0.05), whereas only lactoferrin increased in L13-L16 milk samples. Moreover, the total WAP proportion did not differ between C and DE milks.

**Table 3:** Quantitative analysis of major milk proteins.

	L2-L4		L13-L16	
	C	DE	C	DE
Protein	Mean (sem)	Mean (sem)	Mean (sem)	Mean (sem)
α_s1_-casein	27.60 (0.6) ^a^^,^^c^	25.70 (0.39) ^a^^,^^d^	22.50 (0.32) ^b^^,^^c^	20.80 (0.46) ^b^^,^^d^
α_s2_ -casein	1.30 (0.20) ^c^	1.10 (0.18) ^d^	3.60 (0.18) ^c^	3.50 (0.18) ^d^
α_s2_-like-casein	13.00 (1.18) ^a^	16.80 (0.8) ^a^	12.70 (0.57) ^b^	16.20 (0.35) ^b^
β-casein	26.30 (0.56) ^c^	24.80 (0.71) ^d^	28.60 (0.28) ^c^	27.10 (0.59) ^d^
κ-casein	8.40 (0.28)	7.80 (0.33)	9.00 (0.3)	8.50 (0.15)
**Total Caseins**	**76.60 (0.63)**	**76.00 (0.85)**	**76.40 (0.25)**	**76.00 (0.47)**
WAP	15.40 (0.54)	14.70 (0.55)	15.70 (0.37)	15.60 (0.34)
Lactoferrin	6.00 (0.17)^a^	6.60 (0.20)^a^	5.90 (0.17)^b^	6.50 (0.21)^b^
α-Lactalbumin	0.61 (0.07)	0.41 (0.07) ^d^	0.85 (0.13)	0.76 (0.09) ^d^
Serumalbumin	1.40 (0.14) ^a^	2.10 (0.34) ^a^	1.10 (0.18)	1.40 (0.24)
**Total Whey proteins**	**23.40 (0.63)**	**23.90 (0.25)**	**23.60 (0.85)**	**24.30 (0.45)**

Quantitative results are expressed as a % of total proteins.

^a^ P values <0.05 calculated using the Mann-Whitney test to compare the C and DE groups on days 2–4 of lactation.

^b^ P values <0.05 calculated using the Mann-Whitney test to compare the C and DE groups on days 13–16 of lactation.

^c^ P values <0.05 calculated using the Mann-Whitney test to compare the C milk at L2-L4 and L13-16 days of lactation.

^d^ P values <0.05 calculated using the Mann-Whitney test to compare the DE exposed milk at L2-L4 and L13-16 days of lactation.

When the relative proportions of the main milk proteins were compared between Days 2–4 and 13–16 of lactation in C and DE-exposed rabbits, only α-lactalbumin followed a different pattern (Table 3). While α-lactalbumin remained stable in C milk, its level rose sharply in DE milks (0.041 ± 0.07% *vs*. 0.76 ± 0.09, respectively, *P*<0.05). For α_s2_ and β-caseins, an increase was observed in both the C and DE-exposed groups. Alpha-s1 casein levels fell between L2-L4 and L13-L16 (*P*<0.05).

The relative proportion of phosphorylated isoforms (percentage of the mass signal intensity of each phosphorylation isoform of a given protein relative to the sum of all its phosphorylation isoforms) of α_s1_, α_s2_-like and β-caseins and WAP in C and DE milks was determined at the two stages of lactation (Table 4). On Days 2–4 and 13–16 of lactation, the relative proportions of the less phosphorylated isoforms (1-4P) were higher in DE milk for α_s2_-like-casein (*P*<0.05). However, at the later stage, the most phosphorylated isoform of α_s1_-casein displayed a significant decrease in DE milk (*P*<0.05).

**Table 4:** Phosphorylation of major milk proteins from C and DE rabbits.

		L2-L4		L13-L16	
Proteins	Phosphorylation	C	DE	C	DE
		Mean (sem)	Mean (sem)	Mean (sem)	Mean (sem)
α_s1_-casein	5-6P	9.5 (1.25)	9.4(0.59)	9.8 (0.25)	9.6 (0.22)
	7-8P	16 (1.69) ^b^	14.0 (0.93) ^b^	12.3 (0.46) ^a^^,^^b^	9.7 (0.3) ^a^^,^^b^
α_s2_-like-casein	1-4P	3.5 (0.75) ^a^	7.1 (0.78) ^a^	3.9 (0.7) ^b^	7.6 (0.89) ^b^
	5-8P	8.4 (0.96)	8.3 (1.19)	8.7 (0.26)	7.4 (0.86)
α_s2_ -casein	2-3P	0.5 (0.1) ^c^	0.5 (0.08) ^d^	1.4 (0.07) ^c^	1.3 (0.06) ^d^
	4P	0.8 (0.12) ^c^	0.6 (0.09) ^d^	2.2 (0.15) ^c^	1.9 (0.14) ^d^
β-casein	0-3P	7.2 (0.83) ^c^	7.6 (0.68) ^d^	9.7 (0.08) ^c^	9.6 (0.57) ^d^
	4P	14.8 (0.81)	16.1 (0.67)	15.3 (0.41)	16.2 (0.49)
WAP	0-1P	4.2 (0.77)	3.4 (0.41) ^d^	4.4 (0.26)	4.7 (0.18) ^d^
	2-3P	11.2 (0.62)	11.3 (0.59)	11.3 (0.32)	10.9 (0.39)

Quantitative results are expressed as a % of total proteins.

^a^ P values <0.05 calculated using the Mann-Whitney test to compare the C and DE groups on days 2–4 of lactation.

^b^ P values <0.05 calculated using the Mann-Whitney test to compare the C and DE groups on days 13–16 of lactation.

^c^ P values <0.05 calculated using the Mann-Whitney test to compare C milk at L2-L4 and L13-16 days of lactation.

^d^ P values <0.05 calculated using the Mann-Whitney test to compare DE-exposed milk at L2-L4 and L13-16 days of lactation.

### DE Exposure does not change leptin concentrations in milk

**Fig 5:**
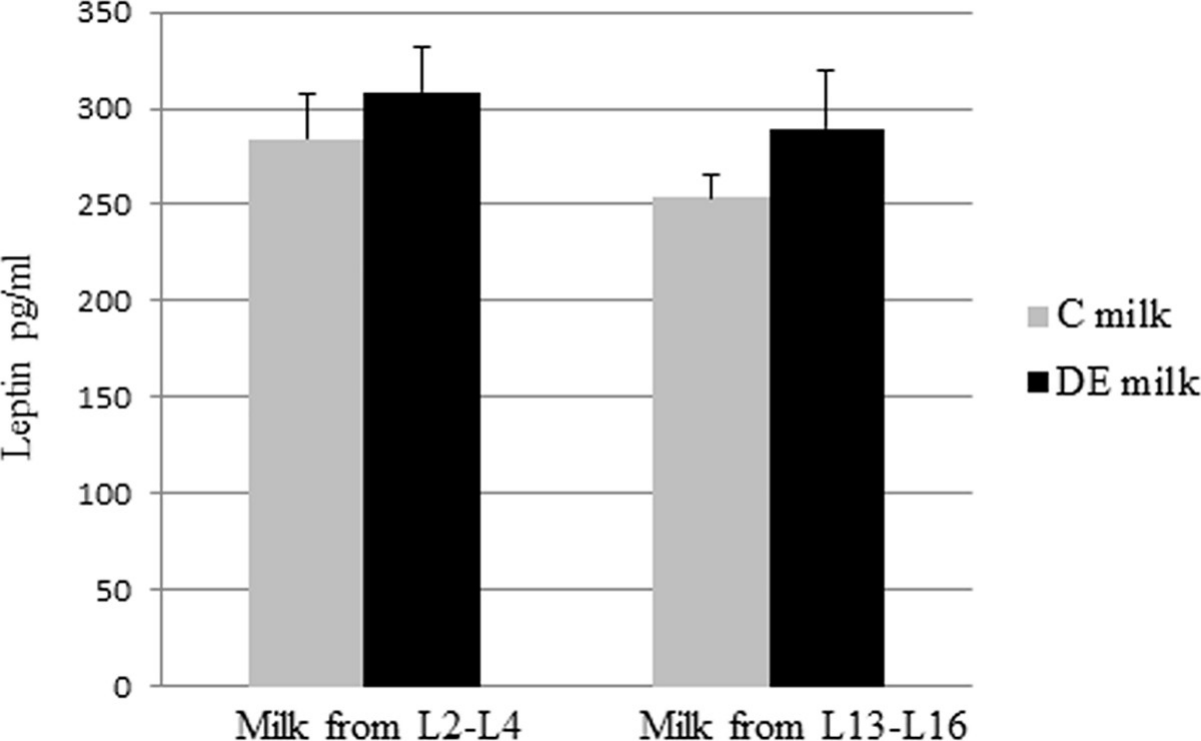
Leptin concentrations in milk from C and DE rabbits on Days 2–4 and 13–16 of lactation. Measurement of leptin levels in milk from control (C) and DE exposed (DE) dams on Days 2 to 4 (L2-L4; 9 C and 9 DE rabbits) and Days 13 to 16 (L13-L16; 9 C and 9 DE rabbits) of lactation. Data are mean ± SEM.

The immunodetection of leptin was performed in whole milk samples collected on days 2–4 and 13–16 of lactation (Fig 5). Leptin concentrations were similar in milk from both C and DE-exposed females at both stages of lactation.

## Discussion

This study revealed the effects on mammary gland development at late pregnancy (Day 28) induced in rabbits exposed to diesel exhaust fumes (1 mg/m^3^, for 2h/day, 5 days/week), throughout pregnancy. This diesel exhaust level was chosen as this experiment was part of a large experimentation aiming at evaluating effect of maternal DE exposure at concentrations close to that of human exposure in large European cities when people drive, ride or walk on big roads twice a day [14]. Exposure to DE did not alter mammary gland histology, but increased the alveolar content in fat globules. By Day 28 of pregnancy, the rabbit mammary gland has entered a differentiated stage of growth [37] after completing the proliferative stage. Although the total surface area occupied by mammary epithelial tissue and lumina was not affected by DE exposure, the relative surface area of fat globules increased dramatically in the mammary lumina of DE-exposed animals. The regulation of adiposity is deemed to be the primary function of leptin, but it also plays a role in other physiological processes, as evidenced by its many sites of synthesis other than fat cells [38]. In particular, mammary epithelial cells produce leptin [38–41], which acts as an autocrine and paracrine factor to influence development and differentiation of the mammary gland [42]. Neither mammary leptin mRNA expression nor protein localisation in mammary epithelial cells differed between C and DE-exposed rabbits at Day 28 of pregnancy. Leptin transcripts, however, tended to be decreased in DE mammary epithelium, suggesting an alteration to mammary lipid metabolism induced by DE. Moreover, when the enzymes involved in fatty acid metabolism were analysed, only the Stearoyl-CoA desaturase (SCD) gene was elevated in DE animals. Stearoyl-CoA desaturase is an enzyme that plays a key role in the composition of fatty acids by generating monounsaturated fatty acids (MUFAs) that provide the principal substrates for the biosynthesis of polyunsaturated fatty acids (PUFAs) and complex lipids such as triglycerides, phospholipids and cholesterol esters involved in energy storage, biological membranes’ composition and signalling molecules [43, 44]. This elevation of Stearoyl-CoA desaturase levels may therefore explain the rise in fat globules observed in the DE-exposed alveolar lumina as well as increased fatty acid content in DE milk during early lactation. During mid-lactation (L13-L16), SFA levels, mainly C10-C12, were higher in DE milk, while PUFA levels were lower, thus suggesting a rebound in the maturation process of the milk. Moreover, since offspring’s weight at birth was decreased in DE rabbits [14], these changes in fatty acid milk composition favouring scSFA, may contribute to the catch-up growth of the pups described in Valentino *et al* [14].

Unlike data obtained in the placenta, we were not able to detect any particles in mammary tissue. In the placenta, non-aggregated and “fingerprint” nanoparticles have been observed at various locations: in the maternal blood space, in trophoblastic cells and in foetal blood, thus demonstrating transplacental transfer [14]. The analysis of mammary sections using electron microscopy did not reveal any accumulation of nanoparticles.

Mammary gland development is a complex process consisting of a series of precise events that are finely regulated by a balance of hormones, growth factors and stromal factors [45]. The mammary gland achieves much of its development during postnatal life, particularly during pregnancy when the lobulo-alveolar structures branch and develop in preparation for lactation. Pregnancy is therefore critical to mammary development. Such periods are highly sensitive to environmental changes that can alter mammary gland structure and function. Most studies to date have been performed using endocrine disrupters and demonstrate that gestational exposure to pollutants such as dioxin, BPA or genistein alter mammary epithelial development [46–48]. Diesel engine exhaust fumes modify the carcinogenic sensitivity of mammary epithelial cells by affecting metabolic activation pathways [49]. The mammary phenotype observed here after DE exposure shares features with a previously described altered mammary phenotype that was induced by an obesogenic diet consumed by rabbits from puberty to pregnancy [30, 31]. During both experiments, the mammary lumina exhibited an increase in their fat globule content. Taking account of the rise in SCD expression, we can therefore hypothesise that exposure to DE and an obesogenic diet may trigger common mechanisms [50]. Human studies have already demonstrated that traffic pollution influences metabolism and is positively associated with elevated BMI rates in children [50, 51].

Although lactation performance was not altered in DE-exposed rabbits at Days 2–4 and 13–16, their milk composition displayed major changes associated with the maternal inhalation of fine particles. Milk from the DE dams exhibited an elevated total fatty acid content resulting from high levels of SFA, MUFA and PUFA. This finding was most likely associated with the *de novo* synthesis of fatty acids in the lactating rabbit mammary gland and might be linked to the increase in mammary SCD expression observed in DE rabbits [52]. The milk lipid composition has been shown to be modified following the ingestion of other pollutants such as bisphenol A [53]. However, few studies have reported on the effects of respiratory pollution apart from an analysis of the consequences of maternal passive smoking in a rodent model, which caused a rise in milk lipid concentrations [54].

In order to take account of any putative effects of diet-associated changes to milk composition we also quantified the main milk proteins, including their predominant phosphorylated isoforms. Although no variations in the total casein ratio were observed in DE milk, a significant increase in α_s2_-like-casein and a significant reduction in α_s1_-casein were determined. Moreover, the relative proportions of less phosphorylated isoforms for αs_2_-like-casein were higher in DE milk. As for total whey proteins, the proportions of both lactoferrin and serum albumin were higher in DE milk. Serum albumin and lactoferrin have antimicrobial activities related to their good binding capacity for metals. They also bind toxic substances, fatty acids and lipopolysaccarides [55, 56]. Therefore, the increase of their concentrations in DE milk during early lactation (L2-L4) may constitute a response to the stress induced by DE inhalation. Moreover, the increase of serum albumin and lactoferrin levels may be related to that of lipid metabolism observed in DE milk.

Altogether, our experiments revealed differences in fatty acid and protein milk composition during early lactation (L2-L4), but these levels were similar when mature milk (L13-16) was examined. Maternal DE exposure throughout pregnancy involves potential risks of growth and metabolic disorders in offspring by affecting foetal and placental development [14]. The consumption of milk during the neonatal period is a characteristic of mammals and provides newborns with essential nutritive support during the transition from neonatal to later life. Milk plays an important role in regulating metabolic programming so that milk composition may have long-term consequences for the health and development of offspring [36, 57]. But although questions remain as to the mechanisms involved in these outcomes, knowledge of early life programming has made significant progress in describing the offspring phenotypes that result from modifications to the neonatal diet. In particular, post-natal brain development is tightly dependent on an optimum fatty acid composition [58]. Moreover, a number of studies have described the very early post-natal period (first ten days) as a critical temporal window that will regulate subsequent food intake in adulthood. Leptin is considered as a key factor in this process [59, 60]. Surprisingly, our study did not reveal any difference in milk leptin levels between the C and DE groups, particularly in milk at early lactation (days L2-L4), a period during which most of the changes to milk composition were observed in our experiments.

It is becoming increasingly clear that developmental exposure to environmental contaminants can have lasting effects on metabolic tissues and have endocrine disrupting potential [61]. A number of studies have proposed the existence of lactational programming [62]. In almost all of them, the effects of pollutants on breast milk composition focused on the ingestion of chemicals such as organochlorine pesticides, bisphenol A, dioxins or heavy metals [46, 53, 63–65]. Few data are available on airway pollution or fine particle inhalation, except in the context of passive smoking [54]. Our experiments are the first to have suggested a link between maternal DE exposure and changes to mammary gland function and subsequent milk composition. It is now of crucial importance to decipher the mechanistic and functional results of DE exposure in mammary epithelial cells, particularly at the level of metabolic pathways. Furthermore, from a public health point of view, such DE exposure-induced changes to milk composition should be taken into account because they may reprogram the developmental trajectory of organs and affect the adult life of offspring.

## Supporting information

S1 Fig Reversed-phase HPLC UV 214 nm chromatograms of rabbit control (C) and DE skimmed milk samples on days 2–4 (L2-L4) and 13–16 (L13-L16) of lactation. Identification of the major milk protein peacks 1: κ-casein, 2: Lactoferrin, 3–4: α_s2_-casein and WAP, 5-6-7: α-lactalbumin and Serum albumin, 8-9-10: α_s2_-like-casein, 11-12-13: α_s1_-casein, 14: β-casein.
(TIF)
