# Supplementary figures and images for: Impact of exposure to diesel exhaust during pregnancy on mammary gland development and milk composition in the rabbit
Source: PLoS One. 2019 Feb 14;14(2):e0212132. doi: 10.1371/journal.pone.0212132 (PMC6375667; doi:10.1371/journal.pone.0212132)

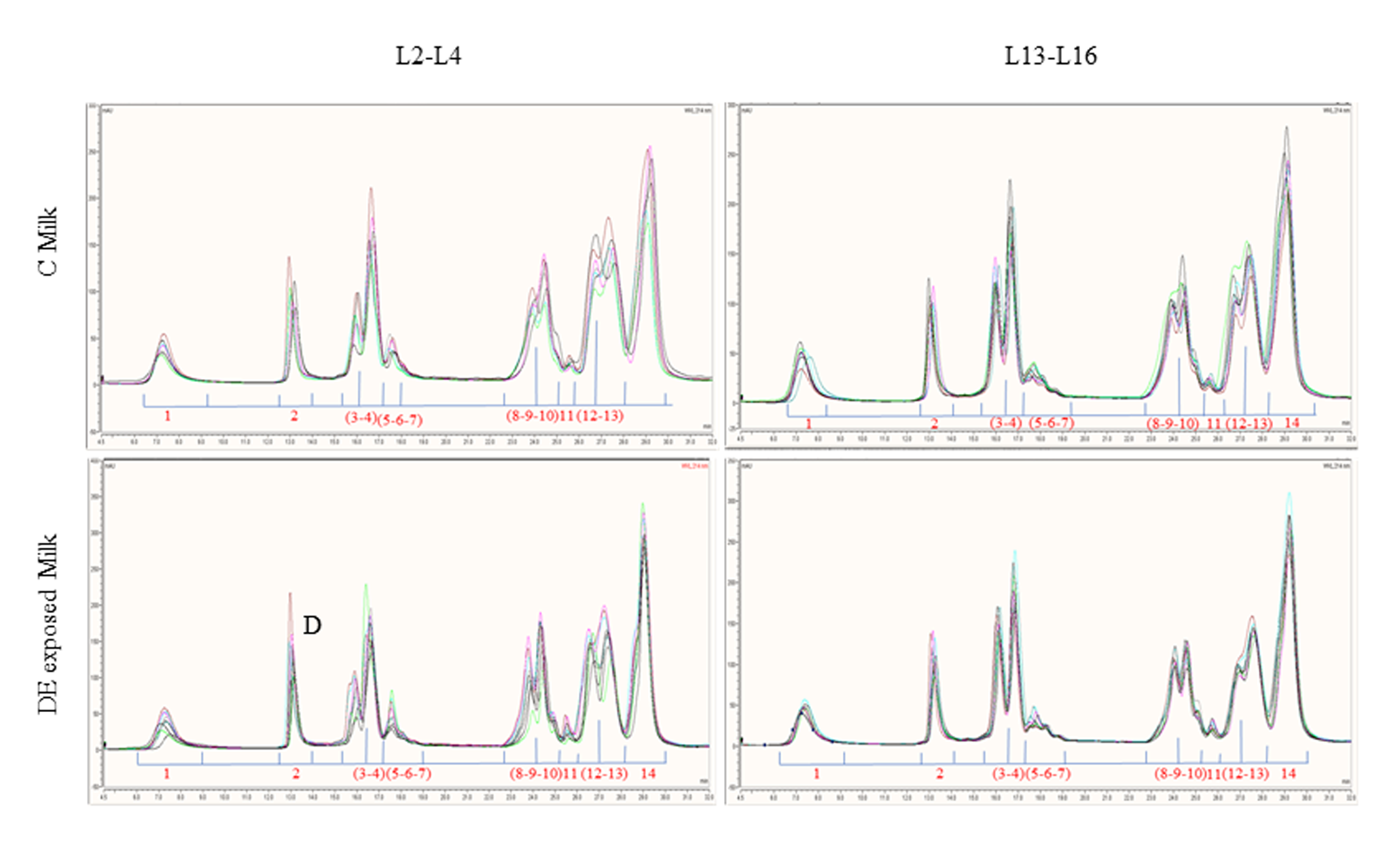

Supplement: S1 Fig — Identification of the major milk protein peacks 1: κ-casein, 2: Lactoferrin, 3–4: αs2-casein and WAP, 5-6-7: α-lactalbumin and Serum albumin, 8-9-10: αs2-like-casein, 11-12-13: αs1-casein, 14: β-casein. (TIF) [file pone.0212132.s001.tif]
